# Humans perceive flicker artifacts at 500 Hz

**DOI:** 10.1038/srep07861

**Published:** 2015-02-03

**Authors:** James Davis, Yi-Hsuan Hsieh, Hung-Chi Lee

**Affiliations:** 1 Computer Science Department, University of California Santa Cruz, 1156 High Street, Santa Cruz, CA, 95033, USA; 2 Department of Computer Science, The University of Texas at Austin, 2317 Speedway, Stop D9500 Austin, TX 78712, USA; 3 Department of Computer Science and Information Engineering, National Taiwan University, No. 1, Sec. 4, Roosevelt Road, Taipei, 10617, Taiwan(R.O.C)

## Abstract

Humans perceive a stable average intensity image without flicker artifacts when a television or monitor updates at a sufficiently fast rate. This rate, known as the critical flicker fusion rate, has been studied for both spatially uniform lights, and spatio-temporal displays. These studies have included both stabilized and unstablized retinal images, and report the maximum observable rate as 50–90 Hz. A separate line of research has reported that fast eye movements known as saccades allow simple modulated LEDs to be observed at very high rates. Here we show that humans perceive visual flicker artifacts at rates over 500 Hz when a display includes high frequency spatial edges. This rate is many times higher than previously reported. As a result, modern display designs which use complex spatio-temporal coding need to update much faster than conventional TVs, which traditionally presented a simple sequence of natural images.

Digital displays are increasing in ubiquity and complexity. Traditional movie theaters and televisions presented a sequence of closely related images at 48–60 Hz. Stereo 3D television presents a coded sequence of frames intended for the left and right eyes at a total of 120 Hz. DLP projection technology creates full color images from a temporal sequence of dozens of very brief monochrome sub-frames, each lasting only microseconds^1^. Rather than a simple sequence of frames, each of which is a natural image, display designers and researchers now think in terms of coding the temporal output of a display. Applications include using three or more sub-frames to allow simultaneous 3D and 2D television^2^, embedding imperceptible codes into normal images^3^,^4^, and making private displays viewable only with the appropriate glasses^5^,^6^.

The light output of modern displays may at no point of time actually resemble a natural scene. Instead, the codes rely on the fact that at a high enough frame rate human perception integrates the incoming light, such that an image and its negative in rapid succession are perceived as a grey field. This paper explores these new coded displays, as opposed to the traditional sort which show only a sequence of nearly identical images.

The key question that must be answered to build these devices is “What framerate is necessary to provide the illusion of a stable picture?” This question has been the subject of research for more than 50 years and nearly all articles and textbooks on the subject contain a statement similar to the following, *The critical flicker fusion rate is defined as the rate at which human perception cannot distinguish modulated light from a stable field. This rate varies with intensity and contrast, with the fastest variation in luminance one can detect at 50*–*90 Hz*^5^,^6^,^7^,^8^,^9^,^10^,^11^,^12^,^13^.

These primary perceptual findings have been incorporated into international standards for display ergonomics^14^, and a belief that “…a frame rate of 72 Hz for computer displays is sufficient to avoid flicker completely.”^15^.

In the present study, we find that viewers can distinguish between modulated light and a stable field at up to 500 Hz, much higher than the widely reported rate. We hypothesize that unconscious rapid eye movements across high frequency edges in the displayed image are responsible.

Most existing studies have been carried out using a spatially uniform light source^16^, however digital displays provide a spatially varying image. Spatio-temporal sensitivity has also been explicitly measured, with reports of a maximum perceivable rate approximately equivalent to spatially uniform lighting^17^,^18^. These studies have sometimes attempted to measure retinal stabilized response, often with special equipment to insure that eye movement does not affect the measurements^19^,^20^. The relationship between eye movement and flicker perception has received mixed reports. Some researchers have reported that eye movement can enhance threshold perception^21^,^22^, while others have concluded that eye movement do not substantially affect the visibility of motion artifacts or flicker on spatial displays^8^,^23^.

The effect measured in this paper is likely due to saccades, and thus related to the phantom array, a repeated pattern observed with high frequency modulation of a bright point or bright line^24^,^25^,^26^. Unfortunately, none of the existing work on phantom arrays addresses the question of the maximum perceivable modulation rate on displays with 2D spatial extent, the information required by display designers.

The work presented here attempts to clarify “the rate at which human perception cannot distinguish between modulated light and a stable field.” We allow for a spatially varying light source and do not attempt to constrain natural eye movements of our subjects. We follow prior work by measuring the viewer's contrast sensitivity, the ratio between background illumination and modulated illumination which is perceivable. However, rather than varying the brightness of the modulated signal as is done in most perceptual research, we mimic real world situations in which displays have constant brightness and the surrounding ambient light level varies. In our tests the display modulates between an image and its inverse at a constant brightness level, while the subject adjusts the level of ambient illumination until flickering artifacts are just noticeable.

## Results

### Uniform Light vs. Spatial Edge

We presented users with a modulated light source, and asked them to determine the level of ambient illumination under which flicker was just noticeable. We performed experiments both with spatially uniform light resembling most prior studies on the critical flicker fusion rate, as well as with a spatially varying image as would be common on display devices such as TVs and computer screens.

In our experiments, uniform modulated light was produced by a DLP projector and consists of a solid “bright” frame followed by a solid “black” frame. The high spatial frequency image is first “bright on the left half of the frame and black on the right”, and then inverted. We observed the effect described in this paper whenever we displayed an image containing an edge and its inverse in rapid succession. The effect was even stronger with more complex content that contained more edges, such as that in natural images. We chose a simple image with a single edge to allow our experimental condition to be as repeatable as possible.

**Figure 1:**
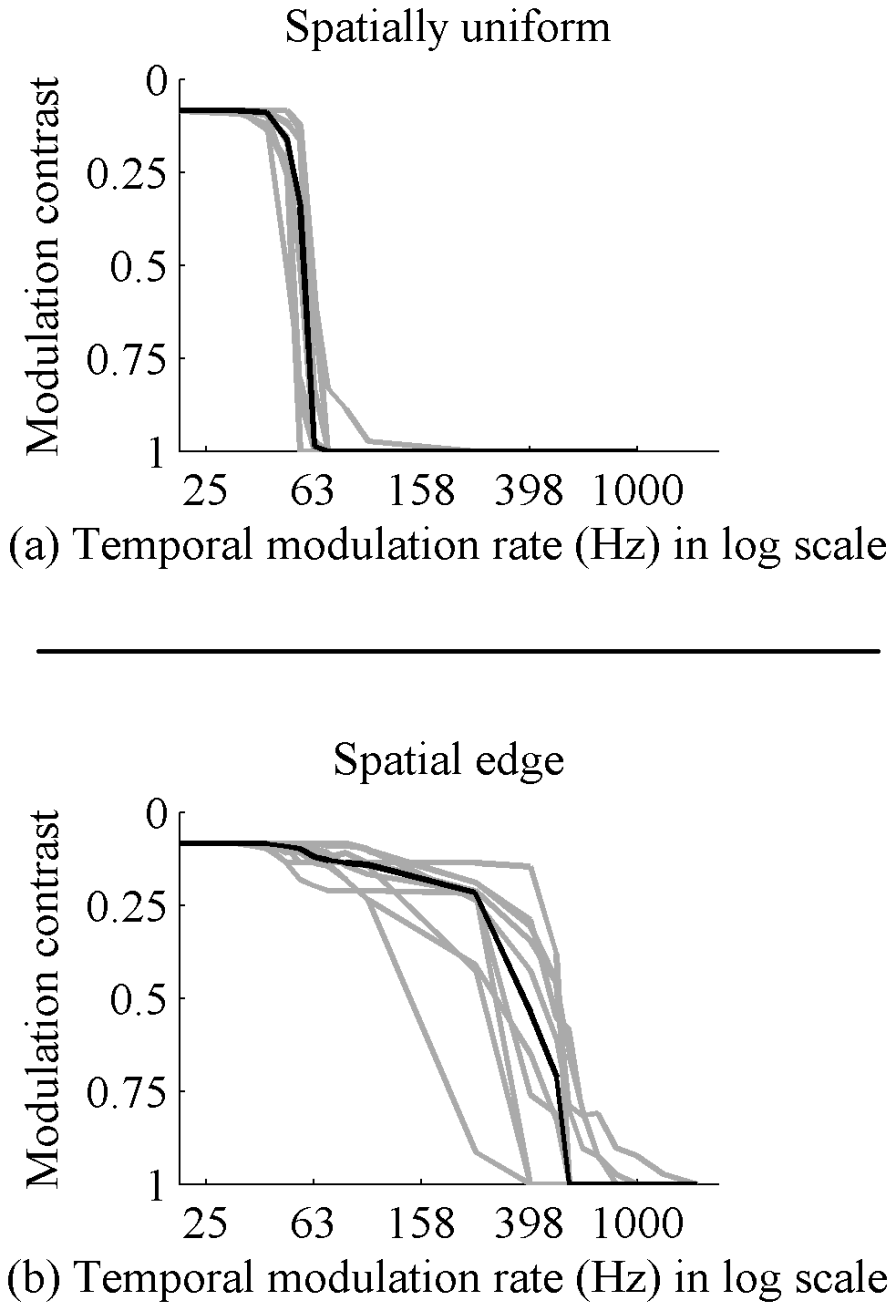
Data collected from ten subjects, together with median sensitivity marked as a darker line. (a) Temporal contrast sensitivity for human observers has previously been reported to drop to zero (the critical flicker fusion rate) near 65 Hz. We duplicate those findings when the modulated light is spatially uniform. (b) When the modulated light contains a high frequency edge in the spatial domain, we measure sensitivity above 500 Hz, much higher than the previously reported rate.

The median contrast sensitivity curve as well as the individual curves for ten subjects is shown in Figure 1. When the modulated light source is spatially uniform, we obtain a contrast sensitivity curve that matches that reported in most textbooks and articles. Sensitivity drops to zero near 65 Hz. However, when the modulated light source contains a spatial high frequency edge, all viewers saw flicker artifacts over 200 Hz and several viewers reported visibility of flicker artifacts at over 800 Hz. For the median viewer, flicker artifacts disappear only over 500 Hz, many times the commonly reported flicker fusion rate.

### Colored light

**Figure 2:**
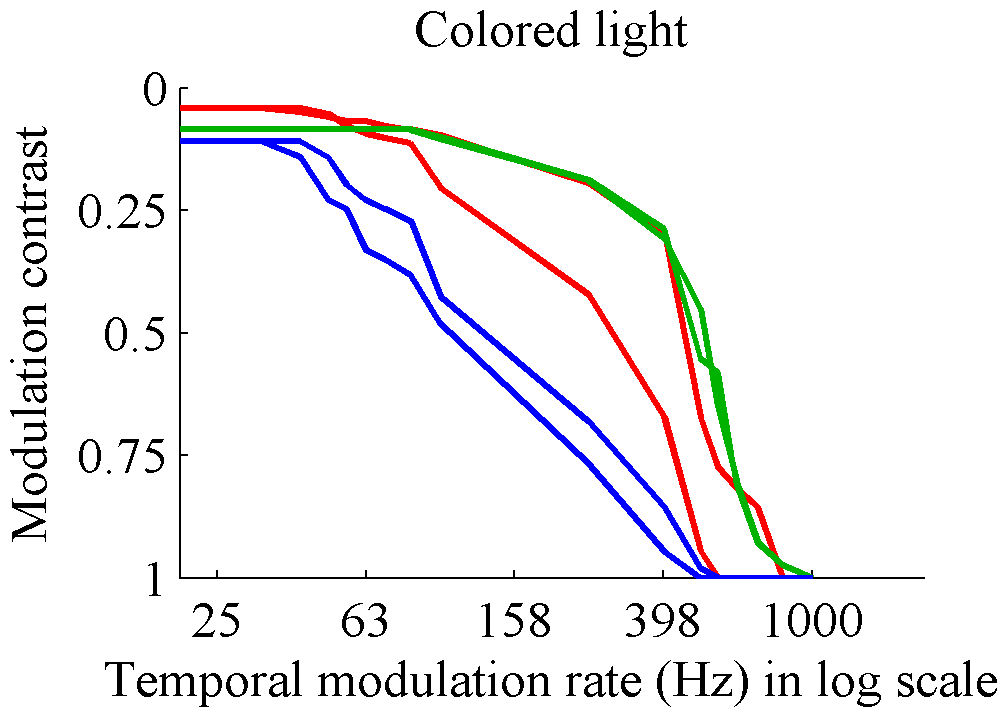
Contrast sensitivity of two subjects when spatial edges were present, using red, green, and blue LED illumination sources. Note that the subjects reported flicker at high modulation rates with all colors, but were most sensitive to green and least sensitive to blue, consistent with past literature on sensitivity.

Our projector allowed for the use of a red, green, or blue light source. In the experiment above, only the green light source was used. To test that the findings were not specific to a particular color, we repeated the experiments with each light source. We used two subjects for this experiment, with all six measurements shown in Figure 2. Note that the subjects reported flicker at high modulation rates with all colors, but were most sensitive to green and least sensitive to blue, consistent with past literature on sensitivity to different wavelengths.

### Spatial frequency

**Figure 3:**
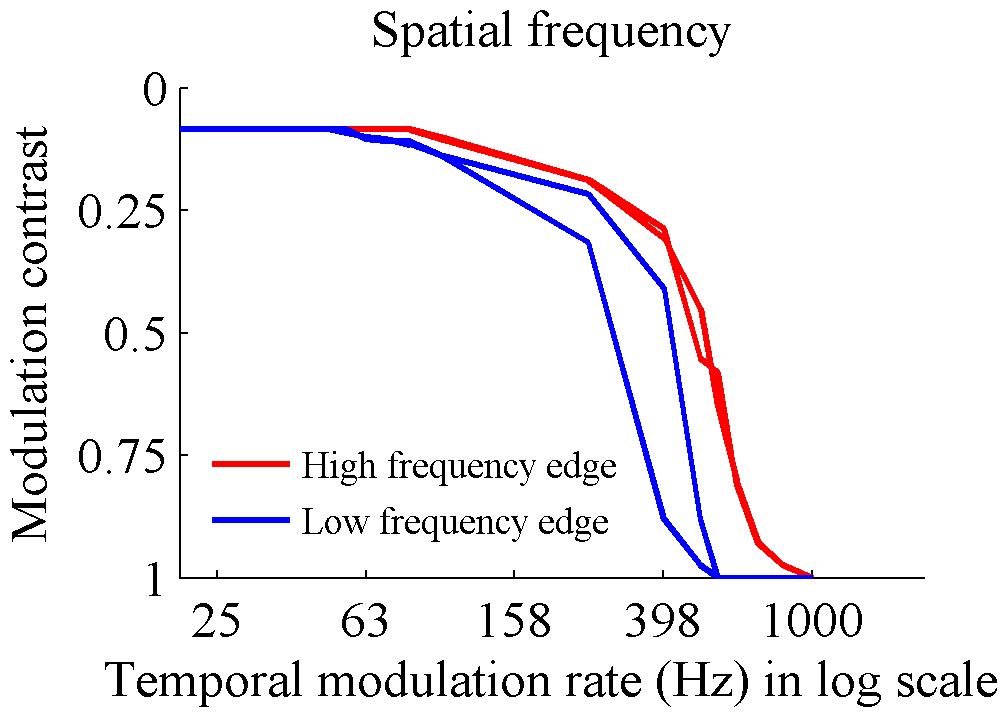
Contrast sensitivity of two subjects when the displayed spatial frequency is reduced by defocusing the projector. Notice that temporal sensitivity is greater for higher frequency spatial content.

We hypothesize that the effect measured in this paper is due to saccades across high frequency spatial edges. If this is true, then we would expect that lower frequency spatial edges would result in lower sensitivity since for a given eye motion at a given retinal receptor the change in modulation pattern would occur at a lower magnitude. We thus repeated our experiments with the projector lens out of focus. This resulted in the edge in the projected image falling from full brightness to black over a distance of 6 mm. We used two test subjects for this experiment, with all four measurements shown in Figure 3. The subjects showed reduced sensitivity to flicker for content with lower spatial frequencies.

## Discussion

The user studies presented in this work provide a critical insight for modern display designers. The flicker fusion rate is much higher than was previously thought for some types of content, and existing international display standards will need to be revised. Existing measurements of critical flicker fusion were driven by the needs of traditional displays. Traditional TVs show a sequence of images, each of which looks almost like the one just before it, and each of these images has a spatial distribution of light intensities that resembles the natural world. The existing measurements of a relatively low critical flicker fusion rate are appropriate for these displays.

In contrast, modern display designs include a sequence of coded fields which are intended to be perceived as one frame. This coded content is not a sequence of natural images that each appears similar to the preceding frame. The coded content contains unnatural sequences such as an image being followed by its inverse. As one example, consider a display designed to provide 3D perception to viewers wearing glasses and 2D perception to viewers not wearing glasses. A very simple display might be built by displaying a sequence of fields which show [Left, Right, 1-Right]. Active shutter glasses that synchronize with the display ensure that the 3D viewer receives the Left and Right sub-fields to the appropriate eyes, with the third field blocked from both eyes. The 2D viewer sees the integral of light from all three fields, equivalent to a contrast reduced version of the Left image. While there are many details of this design neglected here, it contains this new sort of content with images followed by their inverse, so sort of images studied in this work. Our conclusion is that these displays require a higher refresh rate for flicker free display.

It should be noted that in addition to complex coded patterns of content, modern displays have high frequency variations due to modulated backlights and overdrive technology^27^,^28^. These high frequency variations may themselves be content dependant and interact with the effect described here, but are not the focus of this study.

The user studies in this work were carried out with a DLP projector because it provided a variable frame rate for experimentation. However we have observed the effect on every display we have available, including commercial LCD and OLED TV panels. Indeed, the authors first noticed the effect while designing a coded display pattern, and started the present research study in order to explain the unexpected observations. The results presented here now serve as design guidance for our work designing better displays.

## Methods

Modulated illumination was created using a TI DLP Lightcrafter. This projector allows monochrome images to be switched at a user specified rate, up to 4000 Hz. DLP projectors use time multiplexing to produce white light from RGB sub-frames. Since this would be a confounding source of time modulation we enable only one light source in each experiment. Similarly grey-levels are formed from time multiplexing, so we limit our patterns to monochrome 1-bit patterns. The DLP switching rate is on the order of microseconds, much faster than the modulation rates measured here. This effectively produces square wave temporal modulation at the specified rate.

Ambient illumination, the background level in measuring modulation contrast, is created using a Toshiba TLP-X3000 LCD projector. LCD projectors produce grey-levels by physical attenuation of light rather than time modulation. Similarly color is produced by mixing, not time-multiplexing. Our projector uses 3 LCDs so does not contain a spatial color mosaic. We use all three RGB color channels and project grey level images at different intensities. We calibrate the display for each of 255 levels of illumination. We use a darkened room so that the only ambient light is that generated intentionally.

**Figure 4:**
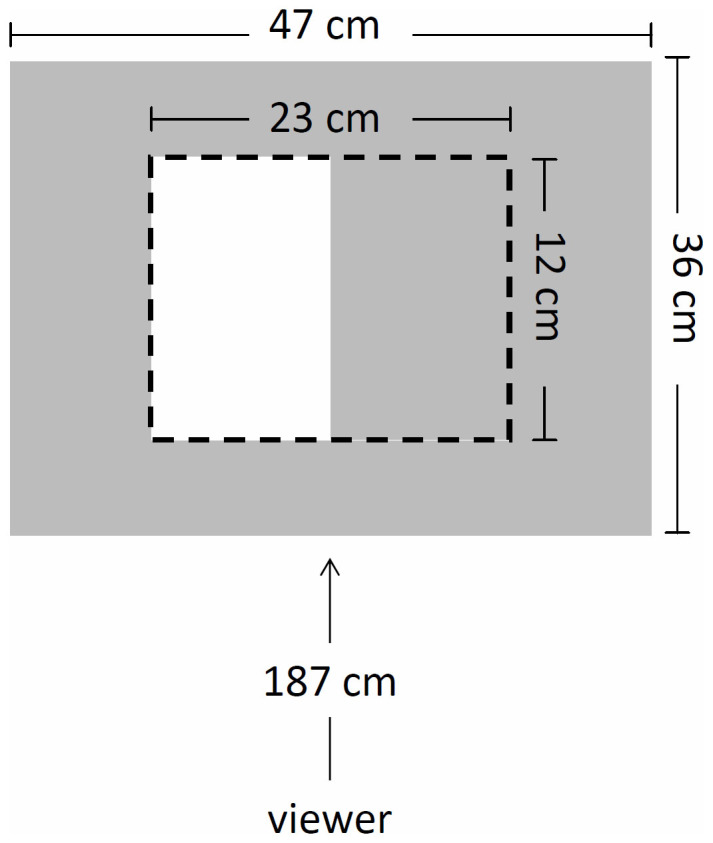
The subject views the modulated illumination projected onto a white wall on top of unmodulated ambient illumination produced by a second projector. The level of ambient illumination is adjusted by the subject until flicker in the modulated area is just noticeable.

The viewer sits 187 cm from a white wall. The modulated light is projected on an area measuring 22.9 cm × 12.4 cm. The ambient illumination extends beyond the modulated illumination, covering an area 47.7 cm × 35.9 cm. The rest of the room is dark. Figure 4 illustrates this configuration.

All light is measured in terms of luminance in units of candela per square meter, cd/m^2^. We use a Sekonic L-758 photometer to measure luminance levels. The modulated light has a measured luminance of 2 cd/m^2^ in the “black” state and 470 cd/m^2^ in the “bright” state. The average luminance is thus 236 cd/m^2^, similar to many commercial displays. The ambient background illumination was user adjustable among 255 possible settings from 20 cd/m^2^ to 2700 cd/m^2^. Thus ambient illumination ranges from 10× dimmer to 10× brighter than the modulated display. Contrast is defined as (L_max_-L_min_)/(L_max_+L_min_), with values in the range 0.95 to 0.09.

Ten subjects are presented modulated light starting from 20 Hz and increasing to 1000 Hz, specifically [20 Hz, 33 Hz, 42 Hz, 50 Hz, 56 Hz, 63 Hz, 71 Hz, 83 Hz, 100H z, 250 Hz, 400 Hz, 500 Hz, 556 Hz, 625 Hz, 714 Hz, 833 Hz, 1000 Hz]. At each tested frequency the subjects were asked to adjust the ambient illumination using computer controls until flicker was just noticeable. The subjects were not given a fixation target and were allowed to look around if desired. A typical user adjusted the ambient level both until it was bright enough that flicker was not visible as well as dark enough that flicker was easily visible before settling into their estimate of the boundary between these conditions. Each subject is tested first with spatially uniform light, and second with spatially varying light. We report the aggregate curve as well as each of the individual curves. The aggregate curve is calculated at each frequency such that 50% of viewers would notice flicker at the specified contrast ratio. Subjects reported in this work were aged 20-29, of both genders, and included two of the authors. This work was approved by the UCSC IRB and was performed in accordance with the approved guidelines. The eight non-authors were informed of and consented to the task, but were not informed of the goals of the experiment until after providing observations. All subjects have normal or corrected to normal vision.

## Author Contributions

J.D. designed the study and wrote sections of manuscript. Y.H. and H.L. have contributed equally to this work: they performed the experiment and wrote sections of manuscript.

## References

[b1] Hornbeck L. J. Digital light processing for high-brightness, high-resolution applications. Proc. SPIE 3013, Projection Displays III. San Jose, CA. 10.1117/12.273880. 27–40 (May 8 1997).

[b2] Scher S., Liu J., Vaish R., Gunawardane P. & Davis J. 3D+2D TV: 3D displays with no ghosting for viewers without glasses. ACM T. Graphic. 32, 21 (2013).

[b3] Cotting D., Naef M., Gross M. & Fuchs H. Embedding imperceptible patterns into projected images for simultaneous acquisition and display. Mixed and Augmented Reality, 2004. ISMAR 2004. Third IEEE and ACM International Symposium on. Arlington, VA, USA. 10.1109/ISMAR.2004.30. 100–109 (2004).

[b4] Raskar R. *et al.* The office of the future: a unified approach to image-based modeling and spatially immersive displays. in Proc. of the 25th annual conference on Computer graphics and interactive techniques. New York, NY, USA. 10.1145/280814.280861. 179–188 (1998).

[b5] Yerazunis W. & Carbone M. Privacy-enhanced displays by time-masking images. in Australian Conference on Computer-Human Interaction. Fremantle, Western Australia. (2001).

[b6] Wu X. & Zhai G. Temporal psychovisual modulation: A new paradigm of information display. IEEE Signal Process. Mag. 30, 136–141 (2013).

[b7] Farrell J. E., Benson B. L. & Haynie C. R. Predicting flicker thresholds for video display terminals. Proc. SID. 28, 449–453 (1987).

[b8] Hoffman D. M., Karasev V. I. & Banks M. S. Temporal presentation protocols in stereoscopic displays: Flicker visibility, perceived motion, and perceived depth. J. Soc. Inf. Display 19, 271–297 (2011). 10.1889/JSID19.3.271 PMC3092720 21572544

[b9] Watson A. B. Handbook of perception and human performance (Wiley, New York, 1986).

[b10] Stidwill D. & Fletcher R. Normal binocular vision: Theory, investigation and practical aspects. (Wiley-Blackwell, Chichester, 2010).

[b11] Wang Y., Ostermann J. & Zhang Y.-Q. Video processing and communications. (Prentice Hall PTR, Upper Saddle River, 2002).

[b12] Foley J. D., Van Dam A., Feiner S. K. & Hughes J. F. Computer graphics: Principles and practice. (Addison-Wesley Professional, Boston, 1996).

[b13] Myers R. L. Display interfaces: fundamentals and standards. (John Wiley & Sons., Chichester, 2002).

[b14] *ISO 9241-3:1992 Ergonomic requirements for office work with visual display terminals (VDTs)-- Part 3: Visual display requirements.* *International Organization for Standardization.* <http://www.iso.org/iso/catalogue_detail.htm?csnumber=16875>, (1992) Date of access: 21/11/2013.

[b15] Barten P. G. J. Contrast sensitivity of the human eye and its effects on image quality. (SPIE, Bellingham, 1999).

[b16] Varner D., Jameson D. & Hurvich L. M. Temporal sensitivities related to color theory. J. Opt. Soc. Am. A 1, 474–81 (1984). 6726494 10.1364/josaa.1.000474

[b17] Robson J. G. Spatial and temporal contrast-sensitivity functions of the visual system. J. Opt. Soc. Am. 56, 1141–1142 (1966).

[b18] Kelly D. H. Spatio-temporal frequency characteristics of color-vision mechanisms. J. Opt. Soc. Am. 64, 983–990 (1974). 4841935 10.1364/josa.64.000983

[b19] Kulikowski J. J. Effect of eye movements on the contrast sensitivity of spatio-temporal patterns. Vision Res. 11, 261–73 (1971). 5579841 10.1016/0042-6989(71)90190-8

[b20] Kelly D. H. Motion and vision. II. Stabilized spatio-temporal threshold surface. J. Opt. Soc. Am. 69, 1340–1349 (1979). 521853 10.1364/josa.69.001340

[b21] Deubel H. & Elsner T. Threshold perception and saccadic eye movements. Biol. Cybern. 54, 351–358 (1986). 3756240 10.1007/BF00355540

[b22] Watson A. B. High frame rates and human vision: A view through the window of visibility. SMPTE Mot. Imag. J. 122, 18–32 (2013).

[b23] Baccino T., Jaschinski W. & Bussolon J. The influence of bright background flicker during different saccade periods on saccadic performance. Vision Res. 41, 3909–16 (2001). 11738456 10.1016/s0042-6989(01)00241-3

[b24] Hershberger W. A. & Jordan J. S. The phantom array: A perisaccadic illusion of visual direction. Psychol Rec. 48, 21–32 (1998).

[b25] Watanabe J., Noritake A., Maeda T., Tachi S. & Nishida S. Perisaccadic perception of continuous flickers. Vision Res. 45, 413–30 (2005). 15610747 10.1016/j.visres.2004.09.010

[b26] Roberts J. E. & Wilkins A. J. Flicker can be perceived during saccades at frequencies in excess of 1 kHz. Lighting Res. Technol. 45, 124–132 (2013).

[b27] Elze T. & Tanner T. G. Temporal properties of liquid crystal displays: implications for vision science experiments. PLoS ONE. 7, 1–20, 10.1371/journal.pone.0044048 (2012). PMC3439495 22984458

[b28] Elze T., Taylor C. & Bex P. An evaluation of organic light emitting diode monitors for medical applications: great timing, but luminance artifacts. Med Phys. 40, 1–6, 10.1118/1.4818056 (2013). PMC3772941 24007183

